# Association Between College Course Delivery Model and Rates of Psychological Distress During the COVID-19 Pandemic

**DOI:** 10.1001/jamanetworkopen.2022.44270

**Published:** 2022-11-30

**Authors:** Abdelrahman ElTohamy, Jessica J. Wang, Justin A. Chen, Courtney Stevens, Cindy H. Liu

**Affiliations:** 1 Harvard Medical School, Boston, Massachusetts; 2 Department of Psychiatry, Brigham and Women’s Hospital, Boston, Massachusetts; 3 Department of Psychiatry, Massachusetts General Hospital, Boston; 4 Department of Pediatric Newborn Medicine, Brigham and Women’s Hospital, Boston, Massachusetts; 5 Department of Psychology, Willamette University, Salem, Oregon

## Abstract

**Importance:**

College students in the US have been heavily affected by the COVID-19 pandemic. In addition to increased rates of depression and anxiety, college students have faced unprecedented stressors, such as geographic relocation and abrupt conversion from in-person classes to online classes.

**Objective:**

To study the association between course delivery model and psychological distress among US college students.

**Design, Setting, and Participants:**

This cross-sectional analysis used national data from the American College Health Association–National College Health Assessment III data set. Data were gathered from a web-based survey administered from January to early June 2021 to full-time US college students attending 4-year programs.

**Exposure:**

Course delivery model was self-reported.

**Main Outcomes and Measures:**

Psychological distress was measured using the Kessler Screening Scale for Psychological Distress.

**Results:**

This study evaluated 59 250 full-time undergraduate students (68.1% women; 51.5% White students; mean [SD] age, 21.2 [4.3] years); 3.5% attended fully in-person classes, 61.2% attended fully online classes, and 35.3% attended a mixed format of in-person and online classes. Students who attended classes fully online reported higher levels of psychological distress than those who attended a mix of online and in-person classes (*b* = 0.76 [99% CI, 0.64-0.88]; *P* < .001). This association remained significant after controlling for geographic region, year in school, gender, race and ethnicity, food security, current anxiety and/or depressive disorders, COVID-19 concerns, and residence (living on campus, off campus with family, or other off-campus arrangements) (*b* = 0.18 [99% CI, 0.04-0.31]; *P* = .001), as well as time spent socializing with friends (*b* = 0.13 [99% CI, 0.002-0.26]; *P* = .009).

**Conclusions and Relevance:**

The findings of this study suggest that mental health professionals may wish to consider the association of course delivery models with mental health outcomes when working with college students. Colleges should be aware of the mental health burden associated with attending fully online classes and consider possible in-person components and supports for students.

## Key Points

**Question:**

Is course delivery model (entirely online vs mix of online and in-person classes) associated with college students’ mental health?

**Findings:**

In this cross-sectional study of a nationwide data set that included 59 250 full-time undergraduate students, those attending fully online classes reported higher levels of psychological distress than students attending a mix of online and in-person classes.

**Meaning:**

The findings of this study suggest that educational institutions and policy makers should weigh the risks and benefits when making determinations regarding school setting and transitions to online classes.

## Teaser

This cross-sectional analysis uses national data from the American College Health Association–National College Health Assessment III data set to study the association between course delivery model and psychological distress among college students.

## Introduction

College students worldwide are facing unprecedented stressors brought about by the COVID-19 pandemic.^1,2^ Many college students have endured the loss of a loved one, faced financial hardships, or experienced racial discrimination during the pandemic.^3^ Rates of depression and anxiety among US college students have increased markedly, with 6 of every 10 college students reporting symptoms of anxiety or depression during the pandemic.^2,4^ Compared with before the pandemic, the prevalence of depressive symptoms among US adults aged 18 to 39 years old during the pandemic more than quadrupled by April 2020.^5^ In addition, most US college students had to relocate from their college campuses^1^ within weeks from the declaration of the pandemic.^6^ A Pew Research Center analysis indicated that between February and July 2020, 2.1 million young adults between 18 and 24 years of age moved back in with their parents.^7^

Concomitant shifts in the learning environment during the pandemic, such as the transition to virtual classes, also altered course delivery models and structures.^8^ During the fall of 2020, 43% of 4-year colleges had fully online classes, 34% included a mix of in-person and online classes, and 13% had fully in-person classes.^9^ Challenges faced by college students in remote learning environments include limited internet or technology access, with negative consequences on academic performance.^10^ Challenges also include the loss of student experiences, such as extracurricular activities, internships, trips to study abroad, service learning, and social events.^10^ The deprivation of these milestone events, as well as the loss of normalcy, friendships, and connection with others, may contribute to personal distress.^11^ In addition, those taking courses online may include students living at home during the first year of the pandemic. Students’ residence—whether with peers or family—may predispose them to different socialization experiences or levels of distress. There is ample evidence that socializing with others is critical for supporting both mental and physical health.^12,13^ However, many mental health professionals may fail to consider the association of such social determinants with mental health outcomes in their clinical approach.^14^

The association between fully online classes and psychological distress—the set of cognitive, emotional, and behavioral symptoms associated with mental health disorders^15^—remains understudied among college students, to our knowledge. One small cross-sectional survey including fewer than 200 participants showed that most college students had difficulties adjusting to online learning and focusing on academic work during the pandemic and that academic challenges were associated with higher rates of depression and anxiety.^16^ Of the studies that have been conducted on the association of online learning with student outcomes, most focused on academic outcomes.^17,18^ To our knowledge, there are no large-scale studies before or during the COVID-19 pandemic that have examined the association between course delivery model (entirely online vs mix of online and in-person classes) and college students’ mental health.

To address this gap, our study analyzed a nationwide sample of undergraduate students in the US from spring 2021 to measure the prevalence of college students who were engaged in course delivery models that were online only, in-person only, and mixed (online and in-person). We examined whether students attending online classes reported higher rates of psychological distress compared with students attending mixed online and in-person classes.

## Methods

### Data Source and Sample

This cross-sectional study was based on the American College Health Association–National College Health Assessment III (ACHA-NCHA),^19^ a biannual survey administered to students in higher educational institutions across the US. The ACHA-NCHA requires institutions either to have all students respond or to randomly select a sample of students. The spring 2021 survey, administered from January to early June 2021, was entirely web-based and included demographic data, psychometric scales, and COVID-19–related questions. Our analysis was based on 59 250 full-time undergraduate students attending 4-year US colleges or universities during spring 2021 with data available on all measures as described. This study followed the Strengthening the Reporting of Observational Studies in Epidemiology (STROBE) reporting guideline for reporting observational studies.^20^ The use of this existing and deidentified data set from the ACHA was approved as an expedited application through the institutional review board at Mass General Brigham. The institutional review board of Mass General Brigham deemed this analysis exempt from human participants review as it used deidentified data from the ACHA-NCHA. Participant consent was first implied when participants clicked on the link within an email message to access the survey, with procedures approved by the institutional review board of the students’ institution. Second, participants were presented with information and instructions on the first page of ACHA-NCHA, including that by clicking “Begin Survey,” they consented to participate in the survey.

### Measures

#### Exposures: Course Delivery Model and Place of Residence

The course delivery model variable was assessed using the following question: “I am taking classes this term,” with response options of “entirely in-person,” “entirely online,” or “a mix of in-person and online classes.” The place of residence was assessed using the following question: “Where do you currently live?” Students who answered “in a fraternity or sorority residence” or “campus or university housing” were coded as the on-campus group. Those who answered “parent/guardian/other family member’s home” were coded as the off-campus with family group. Students who answered “off-campus or other non-university housing” were coded as the other off-campus arrangements group.

#### Outcome: Psychological Distress

Psychological distress was measured using the Kessler Screening Scale for Psychological Distress.^15^ The scale consists of 6 questions, each of which starts with, “During the past 30 days, about how often did you feel…” The questions asked about one’s experience of being “nervous,” “hopeless,” “restless or fidgety,” “so sad nothing could cheer you up,” “that everything was an effort,” and “worthless.” Each question was answered on a 5-point Likert scale, where 0 indicated “none of the time” and 4 indicated “all of the time,” with a total score range of 0 to 24. Higher scores correspond to greater psychological distress. In line with previous studies,^21,22^ psychological distress was examined as a continuous variable in our analysis. The Cronbach α for these items in our sample was 0.89, indicating good reliability.

#### Covariates

A detailed description of our covariates (sociodemographic characteristics, current anxiety and/or depressive disorders, socializing time, and COVID-19 concerns) can be found in the eAppendix in the Supplement.

### Statistical Analysis

We used Stata, version 17 (StataCorp LLC) for our data analysis.^23^ In line with previous research,^3,24^ data cleaning was performed by removing observations with scores outside the plausible range for height (<47.2 inches [120 cm] or >94 inches [239 cm]), weight (<75 lb [34 kg] or >600 lb [272 kg]), and body mass index (>80; calculated as weight in kilograms divided by height in meters squared). Participants who reported not currently having a place to live or temporarily staying with a friend were removed from the analysis (0.1% excluded). Participants with missing data on any of the variables used in the final model were also excluded (5.2% excluded). The resulting sample size was 59 250 full-time undergraduate students in 4-year US colleges or universities. This sample was used for our descriptive analysis in Table 1.

**Table 1.** General Demographic and Key Characteristics of the Sample

Characteristic	Students, No. (%)^a^						
	Total (N = 59 250 [100%])	Course delivery model^b^			Residence^b^		
		In person (n = 2075 [3.5%])	Online (n = 36 273 [61.2%])	Mixed (n = 20 902 [35.3%])	On campus (n = 16 887 [28.5%])	Off campus with family (n = 22 074 [37.3%])	Other off-campus arrangements (n = 20 289 [34.2%])
Region							
Northeast	6932 (11.7)	293 (14.1)	4070 (11.2)	2569 (12.3)	2809 (16.6)	2404 (10.9)	1719 (8.5)
Midwest	13 684 (23.1)	863 (41.6)	4431 (12.2)	8390 (40.1)	6940 (41.1)	1994 (9.0)	4750 (23.4)
South	5304 (9.0)	472 (22.7)	1494 (4.1)	3338 (16.0)	2203 (13.0)	864 (3.9)	2237 (11.0)
West	33 330 (56.3)	447 (21.5)	26 278 (72.4)	6605 (31.6)	4935 (29.2)	16 812 (76.2)	11 583 (57.1)
Age, mean (SD), y	21.2 (4.3)	20.6 (2.9)	21.6 (4.8)	20.6 (3.3)	19.7 (2.0)	20.7 (3.0)	23.0 (5.9)
Current anxiety disorder	11 690 (19.7)	368 (17.7)	6623 (18.3)	4699 (22.5)	3618 (21.4)	3275 (14.8)	4797 (23.6)
Current depressive disorder	9445 (15.9)	264 (12.7)	5441 (15.0)	3740 (17.9)	2874 (17.0)	2613 (11.8)	3958 (19.5)
COVID-19 concerns, mean (SD)^c^	14.0 (5.8)	11.7 (5.8)	14.7 (5.7)	12.9 (5.7)	13.1 (5.6)	14.8 (5.7)	13.9 (5.8)
Race and ethnicity							
American Indian	260 (0.4)	16 (0.8)	139 (0.4)	105 (0.5)	75 (0.4)	74 (0.3)	111 (0.5)
Asian	8804 (14.9)	130 (6.3)	7030 (19.4)	1644 (7.9)	1634 (9.7)	4976 (22.5)	2194 (10.8)
Black	1857 (3.1)	46 (2.2)	1198 (3.3)	613 (2.9)	665 (3.9)	667 (3.0)	525 (2.6)
Hispanic	9061 (15.3)	78 (3.8)	7767 (21.4)	1216 (5.8)	1181 (7.0)	5764 (26.1)	2116 (10.4)
Middle Eastern	598 (1.0)	6 (0.3)	494 (1.4)	98 (0.5)	85 (0.5)	361 (1.6)	152 (0.7)
Multiracial	7634 (12.9)	182 (8.8)	5218 (14.4)	2234 (10.7)	2064 (12.2)	2976 (13.5)	2594 (12.8)
Native Hawaiian	145 (0.2)	2 (0.1)	118 (0.3)	25 (0.1)	20 (0.1)	89 (0.4)	36 (0.2)
White	30 490 (51.5)	1602 (77.2)	14 042 (38.7)	14 846 (71.0)	11 062 (65.5)	7003 (31.7)	12 425 (61.2)
Other^d^	401 (0.7)	13 (0.6)	267 (0.7)	121 (0.6)	101 (0.6)	164 (0.7)	136 (0.7)
Gender							
Men	16 642 (28.1)	694 (33.4)	9588 (26.4)	6360 (30.4)	5138 (30.4)	5859 (26.5)	5645 (27.8)
Women	40 327 (68.1)	1334 (64.3)	25 261 (69.6)	13 732 (65.7)	10 980 (65.0)	15 448 (70.0)	13 899 (68.5)
Other	2281 (3.8)	47 (2.3)	1424 (3.9)	810 (3.9)	769 (4.6)	767 (3.5)	745 (3.7)
International student	3522 (6.1)	87 (4.3)	2422 (6.9)	1013 (5.0)	969 (6.0)	1465 (6.8)	1088 (5.5)
Year in school							
First	14 753 (24.9)	540 (26.0)	8332 (23.0)	5881 (28.1)	7452 (44.1)	5983 (27.1)	1318 (6.5)
Second	12 822 (21.6)	466 (22.5)	7239 (20.0)	5117 (24.5)	4400 (26.1)	4687 (21.2)	3735 (18.4)
Third	15 984 (27.0)	457 (22.0)	10 365 (28.6)	5162 (24.7)	3016 (17.9)	5922 (26.8)	7046 (34.7)
Fourth	12 267 (20.7)	541 (26.1)	7818 (21.6)	3908 (18.7)	1830 (10.8)	3979 (18.0)	6458 (31.8)
Fifth or more	3424 (5.8)	71 (3.4)	2519 (6.9)	834 (4.0)	189 (1.1)	1503 (6.8)	1732 (8.5)
Food security							
High	37 973 (64.1)	1385 (66.7)	23 508 (64.8)	13 080 (62.6)	10 641 (63.0)	15 589 (70.6)	11 743 (57.9)
Low	14 953 (25.2)	509 (24.5)	8832 (24.3)	5612 (26.8)	4507 (26.7)	4800 (21.7)	5646 (27.8)
Very low	6324 (10.7)	181 (8.7)	3933 (10.8)	2210 (10.6)	1739 (10.3)	1685 (7.6)	2900 (14.3)
Socializing time (h/wk)							
Low (0)	5657 (9.5)	70 (3.4)	4459 (12.3)	1128 (5.4)	771 (4.6)	3136 (14.2)	1750 (8.6)
Medium (1-5)	24 350 (41.1)	677 (32.6)	16 268 (44.8)	7405 (35.4)	5309 (31.4)	10 916 (49.5)	8125 (40.0)
High (≥6)	29 243 (49.4)	1328 (64.0)	15 546 (42.9)	12 369 (59.2)	10 807 (64.0)	8022 (36.3)	10 414 (51.3)
Place of residence							
On campus	16 887 (28.5)	1287 (62.0)	5101 (14.1)	10 499 (50.2)	NA	NA	NA
Off campus with family	22 074 (37.3)	146 (7.0)	19 290 (53.2)	2638 (12.6)	NA	NA	NA
Other off-campus arrangements	20 289 (34.2)	642 (30.9)	11 882 (32.8)	7765 (37.1)	NA	NA	NA
Course delivery model							
In-person	2075 (3.5)	NA	NA	NA	1287 (7.6)	146 (0.7)	642 (3.2)
Online	36 273 (61.2)	NA	NA	NA	5101 (30.2)	19 290 (87.4)	11 882 (58.6)
Mixed	20 902 (35.3)	NA	NA	NA	10 499 (62.2)	2638 (12.0)	7765 (38.3)

Abbreviation: NA, not applicable.

^a^ Percentages may not total 100% because of rounding.

^b^ The χ^2^ test and analysis of variance examining characteristics by course delivery model and by residence showed an overall statistically significant difference in distribution (*P* < .001).

^c^ COVID-19 concerns scale is composed of 6 items. Each item was measured on a 5-point Likert scale, where 0 indicated “not concerned at all” and 4 indicated “extremely concerned,” with a total score range from 0 to 24.

^d^ Students who indicated that their racial or ethnic identity is not listed were recoded as “Other.”

Students attending entirely in-person classes were excluded from the regression analyses given their small sample size (n = 2075 [3.5%]). The sample size for our regression analyses was 57 175 students, comprising those who attended fully online classes and those who attended mixed online and in-person classes. The first linear regression model examined psychological distress as an outcome based on course delivery model as an independent variable (block 1). The second model adjusted for covariates through a multiple linear regression analysis (block 2). The third model included the socialization variable (block 3) (Table 1). A simple linear regression model was used for Table 2 block 1. Multiple linear regression models were used for Table 2 blocks 2 and 3. All statistical tests were 2-sided. Consistent with prior published research using ACHA-NCHA data,^25^ a significance level of *P* < .01 and 99% CIs were used given the large sample size and number of comparisons being made.

**Table 2.** Linear Regression Model With Psychological Distress as an Outcome and Course Delivery Model as an Independent Variable (Online Only vs Mixed Format)^a^

Variable	Block 2^b^			Block 3^c^		
	Unstandardized coefficient (99% CI)	Standardized β coefficient	*P* value	Unstandardized coefficient (99% CI)	Standardized β coefficient	*P* value
Region						
Northeast	0 [Reference]	0	NA	0 [Reference]	0	NA
Midwest	−0.03 (−0.22 to 0.17)	−0.002	.72	0.002 (−0.19 to 0.19)	0.000	.98
South	−0.20 (−0.44 to 0.04)	−0.01	.04	−0.164 (−0.403 to 0.075)	−0.008	.08
West	0.114 (−0.06 to 0.29)	0.01	.09	0.07 (−0.10 to 0.24)	0.006	.30
Year in school						
First	0 [Reference]	0	NA	0 [Reference]	0	NA
Second	0.06 (−0.10 to 0.21)	0.004	.33	0.08 (−0.08 to 0.24)	0.006	.18
Third	−0.27 (−0.43 to −0.12)	−0.02	<.001	−0.28 (−0.43 to −0.13)	−0.02	<.001
Fourth	−0.60 (−0.76 to −0.43)	−0.04	<.001	−0.60 (−0.76 to −0.43)	−0.04	<.001
Fifth or more	−0.56 (−0.81 to −0.31)	−0.02	<.001	−0.65 (−0.89 to −0.40)	−0.03	<.001
Gender						
Men	0 [Reference]	0	NA	0 [Reference]	0	NA
Women	0.47 (0.35 to 0.589)	0.04	<.001	0.45 (0.33 to 0.57)	0.04	<.001
Other	2.60 (2.31 to 2.88)	0.09	<.001	2.57 (2.28 to 2.85)	0.09	<.001
Race and ethnicity						
White	0 [Reference]	0	NA	0 [Reference]	0	NA
American Indian	−0.002 (−0.80 to 0.80)	0.000	.995	−0.18 (−0.98 to 0.62)	−0.002	.56
Asian	0.70 (0.54 to 0.86)	0.05	<.001	0.68 (0.52 to 0.84)	0.04	<.001
Black	0.04 (−0.27 to 0.34)	0.001	.76	−0.11 (−0.42 to 0.19)	−0.004	.33
Hispanic	−0.11 (−0.28 to 0.05)	−0.007	.08	−0.23 (−0.40 to −0.07)	−0.02	<.001
Middle Eastern	1.35 (0.83 to 1.87)	0.03	<.001	1.30 (0.78 to 1.82)	0.02	<.001
Multiracial	0.39 (0.22 to 0.55)	0.02	<.001	0.35 (0.18 to 0.51)	0.02	<.001
Native Hawaiian	0.15 (−0.90 to 1.19)	0.001	.72	0.10 (−0.95 to 1.14)	0.001	.81
Other^d^	0.32 (−0.32 to 0.96)	0.005	.20	0.15 (−0.48 to 0.79)	0.002	.54
Food security						
High	0 [Reference]	0	NA	0 [Reference]	0	NA
Low	1.52 (1.39 to 1.64)	0.12	<.001	1.51 (1.39 to 1.64)	0.12	<.001
Very low	3.16 (2.99 to 3.34)	0.18	<.001	3.12 (2.94 to 3.29)	0.18	<.001
Current anxiety disorder	1.03 (0.84 to 1.22)	0.08	<.001	1.05 (0.86 to 1.24)	0.08	<.001
Current depressive disorder	2.76 (2.56 to 2.97)	0.18	<.001	2.72 (2.51 to 2.93)	0.18	<.001
COVID-19 concerns	0.25 (0.24 to 0.26)	0.26	<.001	0.25 (0.24 to 0.25)	0.26	<.001
Place of residence						
On campus	0 [Reference]	0	NA	0 [Reference]	0	NA
Off campus with family	0.30 (0.14 to 0.45)	0.03	<.001	0.12 (−0.03 to 0.28)	0.01	.04
Other off-campus arrangements	−0.54 (−0.69 to −0.39)	−0.05	<.001	−0.61 (−0.76 to −0.46)	−0.05	<.001
Course delivery model						
Mixed	0 [Reference]	0	NA	0 [Reference]	0	NA
Online	0.18 (0.04 to 0.31)	0.02	.001	0.13 (0.002 to 0.26)	0.01	.009
Socializing time						
Low	NA	NA	NA	0 [Reference]	0	NA
Medium	NA	NA	NA	−1.18 (−1.37 to −0.99)	−0.11	<.001
High	NA	NA	NA	−1.73 (−1.92 to −1.55)	−0.16	<.001
*R* ^2^	0.229			0.237		

Abbreviation: NA, not applicable.

^a^ Block 1 was unadjusted (course delivery model variable only). The unstandardized coefficient for online-only relative to mixed format was 0.76 (99% CI, 0.64-0.88), with a standardized β coefficient of 0.07 (*P* < .001) and *R*^2^ = 0.004.

^b^ Adjusted (course delivery model and all single variables except socializing time).

^c^ Adjusted (course delivery model, all single variables, and socializing time).

^d^ Students who indicated that their racial or ethnic identity is not listed were recoded as “Other.”

## Results

This study evaluated 59 250 full-time undergraduate students (68.1% women; 51.5% White students; mean [SD] age, 21.2 [4.3] years). Table 1 summarizes the general demographic and key characteristics of the full sample. More than half the participants (64.1%) reported a high level of food security. Almost one-fifth of the participants (19.7%) reported having a current anxiety disorder, and 15.9% reported having a current depressive disorder.

Of the 59 250 participants in our sample, 3.5% attended fully in-person classes, 61.2% attended fully online classes, and 35.3% attended a mixed format of in-person and online classes. Of the 59 250 participants, 28.5% lived on campus, 37.3% lived off campus with family, and 34.2% lived in other off-campus arrangements. About half the college students (49.4%) reported socializing 6 or more hours a week, while 41.1% of students spent 1 to 5 hours a week socializing. A total of 9.5% of students reported spending 0 hours a week socializing with friends. These rates appear to vary widely based on the various course delivery models. Students attending online-only classes had the lowest socialization levels, with 12.3% of students reporting no socializing compared with 5.4% of students attending mixed format classes and 3.4% of students attending fully in-person classes.

Table 2 presents results of the linear regression models with psychological distress as an outcome and course delivery model as an independent variable (online-only vs mixed-format classes). Compared with the students attending mixed-format classes, those who attended fully online classes reported greater distress (*b* = 0.76 [99% CI, 0.64-0.88]; *P* < .001; block 1). This association remained significant after controlling for region, year in school, gender, race and ethnicity, food security, current anxiety and/or depressive disorders, COVID-19 concerns, and place of residence (*b* = 0.18 [99% CI, 0.04-0.31]; *P* = .001; block 2). Even when controlling for socializing time, the association between attending classes online and increased distress levels remained significant (*b* = 0.13 [99% CI, 0.002-0.26]; *P* = .009; block 3).

Students who lived in other off-campus arrangements reported less distress (*b* = −0.54 [99% CI, −0.69 to −0.39]; *P* < .001; block 2) relative to those who lived on-campus; this association remained significant after controlling for socializing time (*b* = −0.61 [99% CI, −0.76 to −0.46]; *P* < .001; block 3). Students who lived off campus with family reported more distress (*b* = 0.30 [99% CI, 0.14-0.45]; *P* < .001; block 2) relative to those who lived on campus, but this association did not remain significant after controlling for socializing time (*b* = 0.12 [99% CI, −0.03 to 0.28]; *P* = .04; block 3) (Table 2).

## Discussion

Most (61.2%) of the 59 250 US college students in our sample attended classes fully online, 35.3% attended a mixed format of in-person and online classes, and 3.5% attended fully in-person classes. Based on our analyses, it appears that students whose classes were offered entirely online were at risk for increased psychological distress compared with those attending a mix of in-person and online classes. This association remained significant even after controlling for geographic region and a wide range of sociodemographic characteristics, as well as when controlling for students’ reported amount of time spent socializing with friends.

Why might a course delivery model with some in-person experiences be more beneficial than a fully online course delivery model? First, the culture of student life is significantly altered with an online-only format, whereas some degree of normalcy may be preserved with a mixed format that includes at least some in-person experiences. The COVID-19 pandemic’s negative association with school culture formation was a frequently reported concern in a qualitative study of 43 primary and secondary school educators.^26^ Socializing with friends was likely more challenging for those who attended classes only online, as such engagement requires greater intentionality and effort. In contrast, a mixed format still afforded at least some in-person experiences that students were accustomed to, with informal opportunities for social interaction. Relatedly, those who attended classes only online were likely to have altered, limited, or no opportunities for participating in extracurricular activities.^27^ Students who attended classes in a mixed format might also have had the choice of attending online or in-person, affording students the flexibility of attending in the format that is most convenient on any particular day.^28^ Such increased perceived control could also help mitigate the negative effect of stressful situations.^29^

Second, students who attended classes only online may have experienced greater distress from academic challenges.^30,31,32^ Factors other than socialization were likely at play given that the association between course delivery model and psychological distress holds even after controlling for reported socializing time. A 2022 mixed-methods study observed that college and graduate students found it challenging to engage during online classes.^33^ Some of the challenges reported included feeling distracted and procrastinating.^33^ Students may experience decreased motivation to engage with faculty when attending class fully online compared with when there is an opportunity for face-to-face interactions.^34^ Online classes may be held asynchronously, which might also increase the burden of time management, as students would be expected to go through course content on their own.^34^ Teaching methods were likely altered in the transition to online formats; changes in teaching methods were found to be a major source of academic-related frustration among college students during the pandemic.^35^

The shift to online classes was intended to limit in-person contact via social distancing,^36,37^ a key strategy for mitigating COVID-19 transmission.^38^ Despite the protections for physical health afforded by these strategies, our results suggest that fully online classes could be associated with worse mental health. Although a mixed format could present additional logistical complications for educators, our findings suggest that some amount of in-person instructional time may be protective for students’ mental health.

Although our primary study aim was to investigate the association between course delivery model and psychological distress, our secondary analyses revealed an association between students’ place of residence and psychological distress. Students who lived in other off-campus arrangements reported lower levels of psychological distress compared with those who lived on campus. After accounting for socializing time, students living off campus with family did not report different psychological distress levels than those who lived on campus. This finding addresses the current literature that shows mixed findings on the protective or harmful associations between living with family and the mental health of college students. Lee et al^2^ found that more than one-third of students had strained family relationships because of the COVID-19 pandemic, and most of these students found it harder to complete the semester at home. On the other hand, Davitt et al^39^ found that college and university students living with a parent or guardian during the pandemic had less food insecurity, less need to work, lower stress, improved health status, and more home-cooked meals compared with students living on their own. Students who moved residences because of the pandemic, many back to their parents’ homes, were also found to have a greater reduction in alcohol consumption than students who did not move.^40^ Our data indicate that socialization with peers may be an additional factor to consider when assessing how living with family is associated with the mental health of college students.

### Limitations

The findings reported in this study have limitations, including several associated with the study design. First, the nature and extent of the in-person component of the mixed course delivery models are unknown and could vary by school. For example, mixed course delivery models can refer to programs with some online and some in-person components or to programs that have a hybrid model in which some students attend online while others attend in person. On an individual level, mixed course delivery models may also vary. For instance, students who reported having mixed course delivery models may have had one class in person and the rest online, or they may have experienced all classes as hybrid. In addition, some students may have had a choice in the course delivery at any given time. Second, the study’s cross-sectional design means that both the exposure and the outcomes were measured simultaneously, limiting causal inference. Third, the survey was administered online, and the measures were self-reported. Self-reported measures may be affected by recall bias and misinterpretation. Fourth, our study is limited by the available variables in the survey. For example, given the lack of data on socioeconomic status, food security was used as a proxy; however, it may not be an accurate measure of the socioeconomic status of college students. Similarly, while place of residence was included in our analysis, there may have been other factors about college students’ living arrangements that were not considered. In addition, there may be other potentially significant factors associated with college students’ well-being that we did not investigate. Future studies can examine how much of an in-person component is needed when a student engages in a mixed model to offset the mental health cost of online-only classes. In addition, it would be important to understand the extent to which these results hold under various pandemic conditions (ie, during a surge in cases) or under nonpandemic conditions.

## Conclusions

Although the shift to online college classes has been shown to be feasible and arguably necessary in the context of the COVID-19 pandemic,^6,41^ our study suggests a potential negative association between such a shift and college students’ mental health. Our results have implications for educational institutions and policy makers weighing the risks and benefits when making determinations regarding school setting and transitions to online classes. Although online classes may be simpler logistically and may minimize the risk of COVID-19 transmission, they also may increase the risk of negative mental health sequelae that should not be ignored.

Our analysis also offers new insights regarding the association of widely scaled student educational experiences with individual psychological distress. A question that emerges is whether these same results would be maintained if online courses continued for a longer period of time (ie, would adaptations be made by students that would eventually mitigate psychological distress?) or if students had a choice in the way they take their courses. Finally, these results are particularly relevant to mental health professionals within educational settings. Knowing that a student is attending classes fully online may provide insight that informs therapeutic approaches and suggestions for recovery.

## Notes

Supplement. **eAppendix.** Covariates
eReferences.
