# Supplementary material for: Association Between College Course Delivery Model and Rates of Psychological Distress During the COVID-19 Pandemic
Source: JAMA Netw Open. 2022 Nov 30;5(11):e2244270. doi: 10.1001/jamanetworkopen.2022.44270 (PMC9713601; doi:10.1001/jamanetworkopen.2022.44270)
Supplement: Supplement. — eAppendix. Covariates eReferences. [file jamanetwopen-e2244270-s001.pdf]

Supplementary Online Content

ElTohamy A, Wang JJ, Chen JA, Stevens C, Liu CH. Association between college course delivery model and rates of psychological distress during the COVID-19 pandemic. *JAMA Netw Open.* 2022;5(11):e2244270. doi:10.1001/jamanetworkopen.2022.44270

eAppendix. Covariates
eReferences.

This supplementary material has been provided by the authors to give readers additional information about their work.

© 2022 ElTohamy A et al. *JAMA Network Open.*

eAppendix. Covariates

Sociodemographic characteristics

Race/ethnicity, year in school, gender, and geographic region were included as covariates in the analysis. Race/ethnicity was included as a covariate in our analysis given the disparities in COVID-19-related psychosocial stressors between different race groups.[1,2] Race/ethnicity was coded as American Indian, Asian, Black, Hispanic, Middle Eastern, Multiracial, Native Hawaiian, White, or other based on the students' response to the question, "how do you usually describe yourself?" Choices included "American Indian or Native Alaskan," "Asian or Asian American," "Black or African American," "Hispanic or Latino/a/x," "Middle Eastern/North African (MENA) or Arab Origin," "Native Hawaiian or Other Pacific Islander Native," "White," "Biracial or Multiracial," and "My identity is not listed above." Students who reported more than one race were recoded and combined with those who reported "Biracial or Multiracial." Students who reported "my identity is not listed above" were recoded as other. Gender was coded as woman, man, or another gender identity based on students' self-report. Region was coded as Northeast, Midwest, South, or West based on the location of the student's institution. As a proxy for socioeconomic status in college students,[3–5] food security was measured using the 5-item USDA Food Security Short Scale Score with scores ranging from 0 to 6.[6,7] Questions asked about the last 30 days and included items like "I couldn't afford to eat balanced meals." Scores of 0-1 correspond to high or marginal food security, 2-4 correspond to low food security, and 5-6 correspond to very low food security. The Cronbach's α for these items in our sample was 0.86, indicating good reliability.

Current anxiety and/or depressive disorders

© 2022 ElTohamy A et al. *JAMA Network Open.*

Participants who made up these groups reported receiving a diagnosis by a healthcare or mental health professional with either anxiety or depression, *and* had an appointment within the last 12 months to discuss their condition, *and* used medicine and/or therapy or other treatment for their condition. Note that among participants who reported receiving no treatment, they were still included in the respective group if they answered 'no' to "did a healthcare or mental health professional tell you that you do not need treatment, or that you can stop treatment, for [anxiety/depression]?" (a response of "no" to this item reflects individuals who prematurely stopped treatment against medical advice). Individuals without an appointment to discuss their condition within the last 12 months were not included as it was inferred that the condition may not be a present concern. The examples provided to the participants for anxiety disorders include generalized anxiety, social anxiety, panic disorder, and specific phobia. For depressive disorders, the provided examples include major depression, persistent depressive disorder, and disruptive mood disorder.

Socializing Time

Socializing time was measured using the question, "how many hours do you spend in a typical week … socializing with friends." Based on the distribution of responses, students who spent 0 hours socializing with friends were referred to as "low socializers." Individuals who spent 1-5 hours a week socializing with friends were referred to as "moderate socializers." Students who spent 6 or more hours a week socializing with friends were referred to as "high socializers."

COVID-19 concerns

Concerns about COVID-19 were measured using the question, "Over the past 30 days, on average, how much have you been concerned with the following?" The 6 items included in this

variable were, “How long the COVID-19 pandemic will last,” “That you will get COVID-19 [or] That you will get COVID-19 again,” “That someone you care about will get COVID-19,” “Someone you care about will die from COVID-19,” “Not being able to spend time with people you care about,” and “Uncertainty of the future.” Each item was measured on a 5-point Likert scale ranging from “Not concerned at all” with a score of 0 to “Extremely concerned” with a score of 4. The sum score ranged from 0 to 24. The Cronbach’s α for these items in our sample was 0.85, indicating good reliability.

© 2022 ElTohamy A et al. *JAMA Network Open.*

eReferences.

1. ElTohamy A, Hyun S, Macaranas AR, Chen JA, Stevens C, Liu CH. Testing positive, losing a loved one, and financial hardship: Real-world impacts of COVID-19 on US college student distress. *J Affect Disord*. 2022;314:357-364. doi:10.1016/j.jad.2022.07.022

2. Liu CH, Stevens C, Wong SHM, Yasui M, Chen JA. The prevalence and predictors of mental health diagnoses and suicide among U.S. college students: Implications for addressing disparities in service use. *Depress Anxiety*. 2019;36(1):8-17. doi:10.1002/da.22830

3. McLaughlin KA, Green JG, Alegría M, et al. Food insecurity and mental disorders in a national sample of U.S. adolescents. *J Am Acad Child Adolesc Psychiatry*. 2012;51(12):1293-1303. doi:10.1016/j.jaac.2012.09.009

4. Pryor L, Lioret S, van der Waerden J, Fombonne É, Falissard B, Melchior M. Food insecurity and mental health problems among a community sample of young adults. *Soc Psychiatry Psychiatr Epidemiol*. 2016;51(8):1073-1081. doi:10.1007/s00127-016-1249-9

5. Sundermeir SM, Wolfson JA, Bertoldo J, Gibson DG, Agarwal S, Labrique AB. Food insecurity is adversely associated with psychological distress, anxiety and depression during the COVID-19 pandemic. *Prev Med Rep*. 2021;24:101547. doi:10.1016/j.pmedr.2021.101547

6. Blumberg SJ, Bialostosky K, Hamilton WL, Briefel RR. The effectiveness of a short form of the Household Food Security Scale. *Am J Public Health*. 1999;89(8):1231-1234. doi:10.2105/ajph.89.8.1231

7. Economic Research Service, USDA. U.S. Household Food Security Survey Module: Six-Item Short Form. Published online September 2012. https://www.ers.usda.gov/media/8282/short2012.pdf

© 2022 ElTohamy A et al. *JAMA Network Open.*
